# Adenocarcinoma of the colon in a pediatric patient: a rare case report

**DOI:** 10.1093/jscr/rjag451

**Published:** 2026-06-13

**Authors:** Fazeela Bibi, Abuzar Farhad, Abdul Eizad Asif, Haiqa Asif, Hanzala Shahid, Ahmad Sanan, Rameen Zahra, Fatima Hameed, Mishal Tariq, Esham Shahid, Muhammad Abdullah Ali, Janmejay Kumar Singh, Said Hamid Sadat

**Affiliations:** Department of Medicine, Jinnah Medical and Dental College, 22-23 Shaheed-e-Millat Road, Karachi East District, Karachi, 74000, Pakistan; Department of Pediatric Surgery, MTI Khyber Teaching Hospital, University Road, Rahat Abad, Peshawar District, Peshawar, 25120, Pakistan; Department of Medicine, Shalamar Medical and Dental College, Shalimar Link Road, Mughalpura, Lahore District, Lahore, 54840, Pakistan; Department of Medicine, Rashid Latif Medical and Dental College, 35-KM Main Ferozepur Road, Kasur/Lahore District, Lahore, 54000, Pakistan; Department of Medicine, FMH College of Medicine and Dentistry, Shadman Road, Ichhra, Lahore District, Lahore, 54000, Pakistan; Department of Medicine, Khyber Medical College, University Road, Rd No. 2, Rahat Abad, Peshawar District, Peshawar, 25120, Pakistan; Department of Medicine, Shalamar Medical and Dental College, Shalimar Link Road, Mughalpura, Lahore District, Lahore, 54840, Pakistan; Department of Medicine, Shalamar Medical and Dental College, Shalimar Link Road, Mughalpura, Lahore District, Lahore, 54840, Pakistan; Department of Medicine, Shalamar Medical and Dental College, Shalimar Link Road, Mughalpura, Lahore District, Lahore, 54840, Pakistan; Department of Medicine, Shalamar Medical and Dental College, Shalimar Link Road, Mughalpura, Lahore District, Lahore, 54840, Pakistan; Department of Medicine, Khyber Medical College, University Road, Rd No. 2, Rahat Abad, Peshawar District, Peshawar, 25120, Pakistan; Department of Medicine, Teerthanker Mahaveer Medical College and Research Centre, National Highway 9 (Delhi Road), Moradabad District, Moradabad, 244001, Uttar Pradesh, India; Department of Medicine, Kabul University of Medical Sciences “Abu Ali Ibn Sina”, Ata Turk Avenue, Jamal Mena, 3rd District, Kabul, 1006, Afghanistan

**Keywords:** pediatric colon cancer, adenocarcinoma, colorectal malignancy, colonoscopy, signet-ring carcinoma

## Abstract

Pediatric colonic adenocarcinoma remains an extremely rare, aggressive malignancy with non-specific gastrointestinal symptoms. We hereby report a case of a 10-year-old boy who had been suffering from intermittent abdominal pain, constipation, and rectal bleeding for 3 months. Colonoscopy showed an ulcerostricturing lesion in the sigmoid colon, and histology was consistent with poorly differentiated carcinoma with signet-ring cells. There was no metastasis. The child had complete excision of the involved part with an uneventful recovery. This case illustrates the difficulties in diagnosing children with suspected colonic carcinoma and points out the role of early colonoscopy in children with rectal bleeding or changes in bowel habits.

## Introduction

Colorectal carcinoma is one of the most common malignancies in adults but is extremely uncommon in the pediatric population, making up less than 1% of all childhood cancers and 0.3% of all colorectal malignancies [1, 2]. Pediatric colorectal carcinoma differs from its adult counterpart in terms of biological and clinical behavior. It usually presents with symptoms of advanced disease accompanied by aggressive histologic subtypes [3, 4]. According to Karnak *et al*. [1] and Sultan *et al*. [2], children often present with abdominal pain, constipation, and rectal bleeding, which is usually mistaken for benign conditions such as inflammatory bowel disease or parasitic infection, and often leads to delayed diagnosis. Unlike the adult variant, pediatric colorectal cancer (CRC) is poorly differentiated, mucinous, or of the signet-ring cell subtype, which often presents with peritoneal dissemination and poor prognosis [5, 6]. According to Hill *et al*. [7] and Poles *et al*. [8], most pediatric cases are discovered at an advanced stage, with serosal invasion and lymphatic spread already present. In a large multicenter review, Indini *et al*. [5] reported that only 12% of children were diagnosed at Stage I. While hereditary syndromes such as Lynch syndrome and familial adenomatous polyposis are recognized as predisposing factors [9], the majority of pediatric cases arise sporadically. Pediatric tumors are distinguished by their predominantly high mucin content, microsatellite instability, and a lack of KRAS mutations, differentiating them from adult variants [10, 11]. Early colonoscopic evaluation is accordingly considered essential when chronic gastrointestinal bleeding, altered bowel habits, or unexplained anemia occurs in a child. This case report presents a rare case of colonic adenocarcinoma in a 10-year-old boy, highlighting the diagnostic approach and clinical reasoning in the management of pediatric patients with chronic gastrointestinal symptoms.

## Case description and investigations

**Figure 1:**
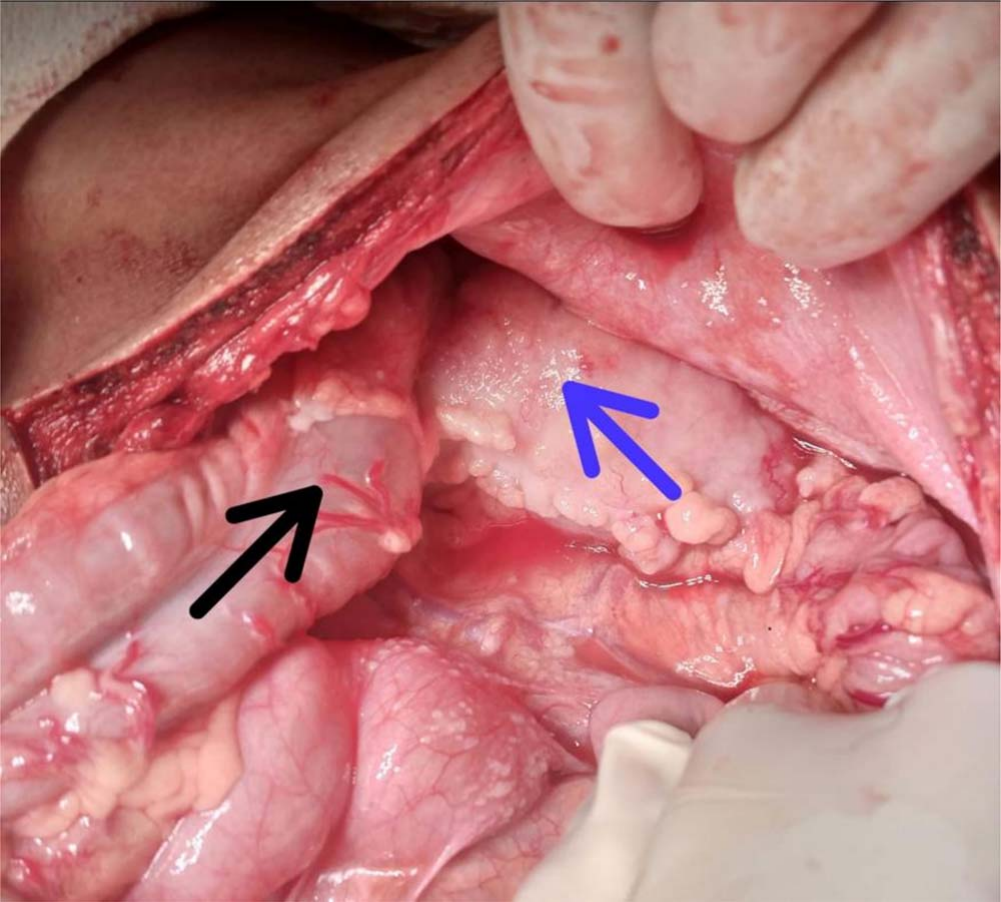
Intraoperative view during exploratory laparotomy showing the transverse colon (black arrow) and the mass (blue arrow) consistent with adenocarcinoma involving the colon in a pediatric patient undergoing segmental resection.

The patient is a 10-year-old male who has been experiencing symptoms of intermittent abdominal pain, constipation, and rectal bleeding for the last three months. There was no loss of weight, vomiting, or family history of malignancy. General examination revealed a soft, mildly distended abdomen, and per rectal examination showed soft, blood-stained stools without any polyps or fissures. There were no abnormalities in vital signs. Plain X-ray of the abdomen revealed a distended bowel with air-fluid levels, indicating partial intestinal obstruction. Examination under anesthesia revealed blood-staining without frank fissures, bleeding due to hemorrhoids, or rectal polyps. Colonoscopy revealed a friable ulcerative stricture in the sigmoid colon about 25 cm away from the anal verge, along with biopsy. Other investigations, including blood counts and coagulation studies, were within normal limits. Hepatitis B surface antigen was negative, and there was no anti-HCV antigen. Peritoneal fluid examination for cytology revealed occasional mesothelial and inflammatory cells without any malignant cells, suggesting a benign exudative effusion. HRCT examination of the chest revealed normal lung fields without any lymphadenopathy or pulmonary nodules, suggesting the absence of metastasis. Histopathological examination of the rectal biopsy revealed poorly differentiated adenocarcinoma with signet-ring cells. The final diagnosis was colonic adenocarcinoma. Exploratory laparotomy confirmed a friable ulcerative growth in the sigmoid colon without any serosal invasion or nodal involvement. Segmental resection of the adenocarcinoma (as shown in Fig. 1) with primary anastomosis was carried out. The patient made an uneventful post-operative recovery and was subsequently discharged on the sixth post-operative day. He remains symptom-free on follow-up without any evidence of recurrence to date. In this case, a differential diagnosis included intestinal lymphoma; intestinal lymphoma is a malignancy that occurs in children frequently and may produce similar symptoms due to intestinal obstruction. Other causes of intestinal obstruction, such as intussusception, Hirschsprung’s disease, Meckel’s diverticulum, and congenital bands, were excluded.

## Discussion

Colorectal carcinoma in children differs from its adult counterpart in both clinical presentation and tumor biology. It is often diagnosed late because its initial manifestations are frequently attributed to benign causes [1, 2, 4]. Most patients had symptoms for several months before diagnosis, and more than 80% presented with locally advanced or metastatic disease [3]. Similarly, it is noted that signet-ring morphology was present in 45% of pediatric cases, significantly higher than in adults, and correlated with worse outcomes [7]. Histologically, mucinous and signet-ring cell adenocarcinomas are predominant in children, leading to early peritoneal dissemination and poor prognosis [5, 12]. At the same time, other works emphasized that these histologic subtypes often present without a family history or predisposing condition, suggesting a unique pathogenic pathway distinct from hereditary syndromes [4, 12]. Nevertheless, germline mutations associated with Lynch syndrome and mismatch repair defects remain important considerations in young patients and warrant genetic counseling [9]. Early colonoscopy plays a pivotal role in diagnosis, as imaging alone is often nondiagnostic [10, 11]. Pediatric CRC is rare, and its aggressive nature makes early endoscopic assessment crucial for prognosis. Once diagnosed, surgical resection with clear margins remains the mainstay of treatment [10]. In this case, segmental colectomy achieved complete resection, and the patient remains disease-free at follow-up. Adjuvant chemotherapy is typically reserved for node-positive or metastatic disease. However, its benefit in pediatric patients remains less defined due to the scarcity of prospective data [8]. Differential diagnosis in pediatric intestinal obstruction must also include lymphoma, which accounts for the majority of gastrointestinal malignancies in children and may present with similar obstructive or bleeding symptoms [6]. Additionally, mechanical causes such as intussusception or congenital adhesions should be considered early, although persistent bleeding should prompt further endoscopic investigation [13]. Overall, pediatric CRC carries a poorer prognosis than adult disease, with five-year survival rates ranging from 20% to 40% depending on stage at diagnosis [7, 8, 12]. Early suspicion, prompt colonoscopic biopsy, and multidisciplinary management remain critical to improving survival outcomes in this rare but lethal malignancy.

## Conclusion

Pediatric colorectal adenocarcinoma is a rare condition that tends to have a late presentation. Biopsy in children who have rectal bleeding or abdominal pain needs to be done immediately. The primary treatment that has been found to work effectively in pediatric colorectal cancer is surgery. Increased awareness in the medical field can help make the treatment of pediatric colorectal cancer more effective.
